# Sex Differences in Sociability for *Drosophila* *melanogaster* with Altered Gut Microbiomes

**DOI:** 10.17912/micropub.biology.001135

**Published:** 2024-02-27

**Authors:** Peyton Panos, Tony Chan, Nayana Gowda, Jeffrey Hong, Rhea Kansal, Katherine Shen, Emily McLean

**Affiliations:** 1 Division of Natural Sciences, Oxford College of Emory University, Oxford, GA USA

## Abstract

In *Drosophila melanogaster* the gut microbiome has been shown to influence multiple behaviors, including aggressive social behavior. Here, we investigate the effect of the *Drosophila* microbiome on pro-social behavior. We predicted that reducing the microbiome would lead to a decrease in pro-social behavior in adult flies. After altering the flies’ microbiomes, we observed that virgin male flies with reduced microbiomes were significantly less social than virgin male control flies (t=3.09, p=0.006). We did not observe this difference in virgin female flies (t=0.344, p=0.73), or mated flies of either sex (males: t=0.456, p=0.66; females: t=0.271, p=0.79). Our results suggest that the role of the Drosophila microbiome in pro-social behavior is dependent on both sex and previous social experience.

**Figure 1. The microbiome impacts sociability, but not activity, in male virgin Drosophila:**
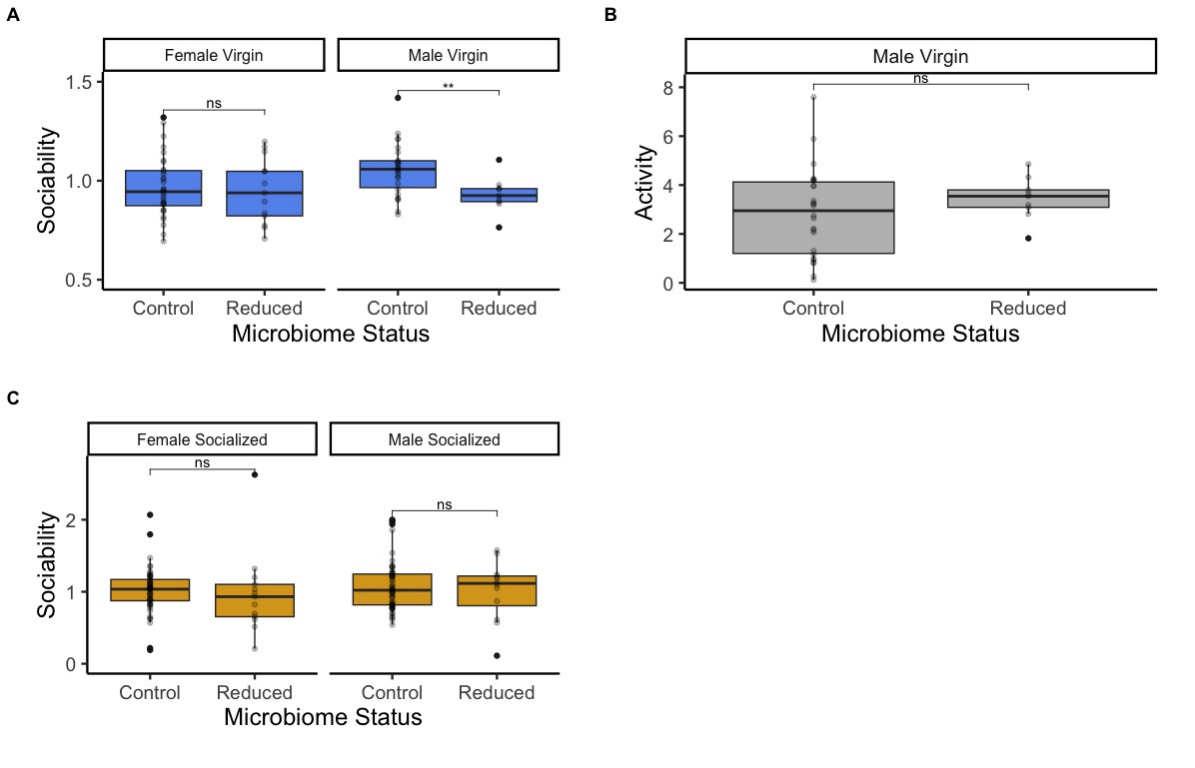
A) **Male virgin flies with a reduced microbiome are less sociable than male virgin flies with a control microbiome, but this pattern is not observed in females.** Female flies with control and reduced microbiomes showed no difference in sociability (t=0.344, p=0.73), while male flies with reduced microbiomes were less social than male flies with control microbiomes (t=3.09, p=0.006). Sociability scores were determined by how often flies were found in the same quadrant of a divided petri dish (see Methods). We conducted 45 female trials, 32 using flies with control microbiomes and 13 using flies with reduced microbiomes. We conducted 35 male trials, 26 using flies with control microbiomes and 9 using flies with reduced microbiomes. B) **Microbiome status does not influence the activity level of male virgin flies.** The y-axis shows “Activity” as measured by quadrant crossings per minute. We found no significant difference in activity levels between male virgin flies with control (n=26) and reduced microbiomes (n=9, t=-1.29, p=0.21). C) **Microbiome status does not influence the sociability of previously socialized flies.** Socialized female flies with control and reduced microbiomes showed no difference in sociability (t=0.271, p=0.79). Socialized male flies with control and reduced microbiomes also showed no difference in sociability (t=0.456, p=0.66) We conducted 66 female trials, 53 using flies with control microbiomes and 13 using flies with reduced microbiomes. We conducted 66 male trials, 54 using flies with control microbiomes and 12 using flies with reduced microbiomes.

## Description

The gut microbiome is a complex community of microorganisms which has been shown to influence behavior in humansand other organisms (Ezenwa et al., 2012) . In *Drosophila,* the gut microbiome has been shown to influence sleep (Silva et al., 2021) , egg laying (Qiao et al., 2019) , and mating success (Jia et al., 2021) , as well as aggressive social behavior. In male *Drosophila* there is a marked decrease in aggressive behavior and mating success with the removal of the gut microbiome (Jia et al., 2021) , although, interestingly, this seems to be dependent on methodology as male flies treated with antibiotics appear to be more aggressive than control flies (Grinberg et al., 2022), whereas male flies grown from sterilized embryos show reduced aggression (Jia et al., 2021) .

Given the wide variety of effects the microbiome has been shown to have on fruit flies, we were curious about how the microbiome may influence non-aggressive social behaviors (sociability). Sociability is a measure of the degree to which members of the same species participate in non-aggressive interactions. In *Drosophila* there are several types of non-aggressive social interactions, such as congregation on food for feeding or egg-laying. Both adult flies and larvae are attracted to volatiles from food substrates that have previously been occupied by larvae and this attraction appears to be mediated by volatiles emerging from the larval microbiome (Venu et al., 2014).

Sociability in adult flies is an emerging phenotype of interest (Scott et al., 2018; Sun et al., 2020) . Previous studies have quantified by sociability as a measure of how much often flies occupy a shared portion of a divided behavioral testing chamber. This phenotype appears to have a genetic basis and to be influenced by both sex and previous social experience. Male flies tend to be more sociable than female flies, and flies that have been isolated tend to be less sociable than flies with previous social experience (Scott et al., 2018) .

In this study, we investigated how reducing the Drosophila microbiome impacts sociability of males and females, in both virgins and socialized flies *.* We hypothesized that if the microbiome of adult flies is producing attractive volatiles, that reduced microbiome flies would have reduced sociability as compared to control microbiome flies.

To test this hypothesis, we generated flies with control and reduced microbiomes and housed them for 2-7 days in isolation or in mixed sex social groups (see Methods). We then transferred the flies to behavioral testing chambers where we measured the sociability of groups of four flies of the same sex and social condition (isolated or socialized). The isolated flies were collected as virgins and remained so throughout behavioral testing.

We found that there was a sex disparity in the changes to sociability for treated and untreated flies and that our hypothesis was supported, but only for male virgin flies. Between virgin flies with control and reduced microbiomes, the sociability of female fruit flies was relatively unchanged. On the other hand, the virgin male fruit flies with control microbiomes were more sociable than virgin male flies with reduced microbiomes ( Figure 1A ). The difference in sociability between virgin males with control and reduced microbiomes appears to be independent of any differences in activity levels between these conditions. Previous work has indicated that activity and sociability are not correlated (Scott et al., 2018) , and we did not see any significant differences in the activity levels of male virgin control and reduced microbiome flies ( Figure 1B ). Interestingly, in previously socialized flies, microbiome reduction did not alter sociability for male or female flies ( Figure 1C ). Taken together, our results suggest that the way that the microbiome affects sociability may differ between male and female fruit flies. The impacts of the microbiome on sociability may also differ between previously isolated and previously socialized flies.

Previous work has also identified sex differences in sociability, with male flies generally being more sociable than females (Scott et al., 2018) . Our results indicate that male flies may also be more sensitive to the presence of a microbiome than female flies. Further work is necessary to determine if these differences are due to olfactory processes, differences in the gut-brain axis of male and females or other mechanisms. Our finding that virgin males are less sociable when microbiomes are reduced, but experienced males are not, suggests that the role of microbiome in sociability may be plastic or dependent on previous social experience.

Future work may address new questions raised by our findings. For instance, is sociability shaped by individual effects of bacterial metabolism within the flies and/or does the microbiome influence inter-fly chemical signaling? Furthermore, is there a specific microbial composition that is associated with increased sociability, and subsequently are there microbiomes which are associated with a decrease in sociability? Our study was not able to address the role of anaerobic microbes or the consequences of completely removing the microbiome – these are also open questions for future work. Though mechanistic questions remain, these data point to a clear difference in how male and female *D. melanogaster* alter their social behavior in response to their microbial communities. This difference, and the questions it generates, may provide new insight into sex differences in the evolutionary role of sociability.

## Methods

*Fly stocks and maintenance*

*Drosophilia melanogaster* was cultured on Carolina Biological Instant Fly Food using standard techniques. CantonS and 28127/DGRP-42 line from the Drosophila Genetic Reference Panel were used in this experiment, both ordered from the Bloomington Drosophila Stock Center at Indiana University. CantonS and 28127/DGRP-42 showed no difference in sociability, so their data was pooled for this analysis. Sociability assays were always conducted using four flies of the same genotype.

*Generation of treated and control flies*

Approximately 50 adult flies were transferred from their stock vial into an egg collection cage. After 24 hours, eggs were removed from the egg collection cage and, in a sterile hood, transferred to a 15mL filter tube. The filter tube was a 15mL centrifuge tube where the bottom had been cut off with a hacksaw and replaced with a 20nm nylon mesh filter. To generate treated flies, the eggs in the filter tube were suspended in a 1% sodium hypochlorite solution for 5 minutes. Then, the filter tube was transferred into 70% ethanol for 1 minute and sterile water for one minute. These washes were repeated twice. After the wash steps, the eggs were transferred to a standard fly food vial filled with autoclaved Carolina Biological Instant Fly Food. Sterile water was added to rehydrate the food. To generate control flies, the same process was used, but the initial 5 minute soak was done in sterile water, instead of sodium hypochlorite. Vials were incubated at 27C until eclosion.

*Verification of microbiome status*

When adults emerged, we determined the success of the treatment by homogenizing three adult flies from each vial in LB broth, plating the homogenate on LB agar and incubating at room temperature for 48 hours. Fly homogenates which produced colonies indicated the presence of a microbiome and flies that emerged from the same vial were subsequently labeled as control microbiome flies. Fly homogenates which did not produce colonies indicated the absence of aerobic microbes, but we were unable to determine if obligate anaerobes or intracellular parasites were present. Flies that emerged from the same vial as the homogenates which did not produce colonies were subsequently labeled as reduced microbiome flies. Adult flies were collected and housed for 2-7 days before behavioral trials were conducted. Virgin flies were collected within 8 hours of eclosion and housed in isolation before behavioral testing. Socialized flies were collected within 24 hours of eclosion and housed in mixed sex groups of 8 flies before behavioral testing. Flies were incubated at 25C and on a 12h light dark cycle from collection to behavioral testing.

*Sociability assays*

Four flies of the same sex and social experience condition (virgin/socialized) were placed in a behavioral testing chamber, using very light cold anesthesia. No fly had previous exposure to any of the other flies in its behavioral testing chamber. Virgin flies had previous been held in isolation, and only one fly from any mixed sex group was included in any behavioral testing chamber. Each behavioral testing chamber was a single 30mm petri dish, which was divided into quadrants by a 3D printed insert that had a small opening between each quadrant. Behavioral trials were recorded by video camera. Flies were given two hours to recover from cold anesthesia and acclimate to the testing chamber, then fly positions were measured every 5 minutes for 4 hours. Sociability scores were determined by the number of flies sharing a quadrant. Specifically, we followed Scott et al. (2018) and counted the number of flies in each quadrant and took the variance of this set of numbers as the sociability score for that timepoint. We averaged the sociability scores across the entire four hours of recording to assign a sociability score for each trial.

*Determination of activity levels of virgin male flies*

To investigate the potential role of fly movement in sociability scores, we measured the activity level of our male virgin fly trials by counting the number of times a fly crossed a quadrant barrier. We counted all quadrant crossings in the first 90 minutes after the 2 hour acclimation period.

## Reagents

**Table:** 

**Strain/Stock Number**	**Genotype**	**Available from**
CantonS/64349	Wild-type	Bloomington Stock Center
DGRP-42/28127	Wild-type	Bloomington Stock Center
